# Supplementary material for: Simulation of Air Puff Tonometry Test Using Arbitrary Lagrangian–Eulerian (ALE) Deforming Mesh for Corneal Material Characterisation
Source: Int J Environ Res Public Health. 2019 Dec 19;17(1):54. doi: 10.3390/ijerph17010054 (PMC6982245; doi:10.3390/ijerph17010054)
Supplement: Supplementary file 1 [file ijerph-17-00054-s001.zip › ijerph-612404-SI.pdf]

International Journal of
Environmental Research and Public Health

Supplementary material

Manuscript ID: ijerph-612404

Title: Simulation of Air Puff Tonometry Test Using Arbitrary Lagrangian-Eulerian (ALE) Deforming Mesh for Corneal Material Characterisation

Authors: Osama Maklad *****, Ashkan Eliasy, Kai-Jung Chen, Vassilios Theofilis, Ahmed Elsheikh

International Journal of
Environmental Research and Public Health

MDPI

Mesh independence study

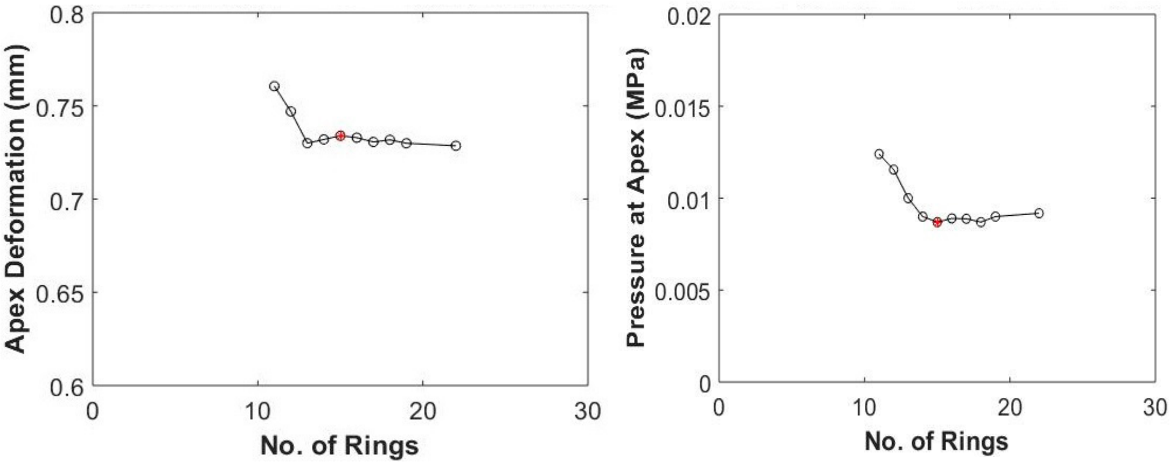

Figure S1. Mesh dependence study on both domains of the model, showing the apex deformation from the eye model and pressure on apex from the CFD model.

International Journal of
*Environmental Research
and Public Health*

MDPI

CorVis-ST internal CFD model

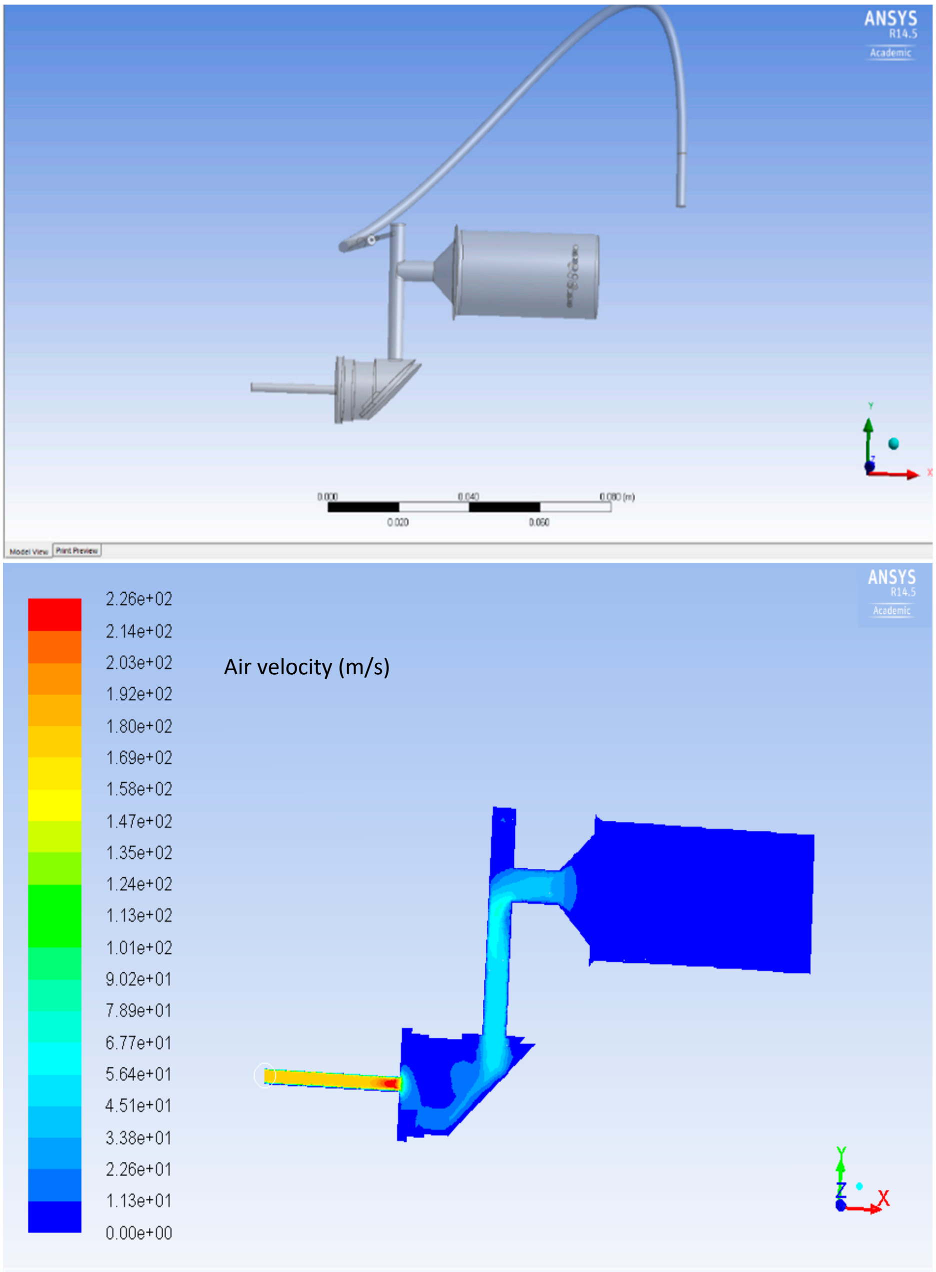

Figure S2. CorVis-ST CAD drawing as supplied from the manufacturer and a CFD solution of the air velocity inside the device starting from the piston at 2m/s ending at the nozzle at 168 m/s
